# OsHOX24 Modulates Grain Size and Salt Tolerance Via OsPP2C09 in Rice

**DOI:** 10.1186/s12284-026-00920-w

**Published:** 2026-06-11

**Authors:** Bin Gao, Shibo Li, Zhixue Huo, Leqin Chang, Jing Ning, Wei He, Liang Luo, Mengna Mo, Yuxing Zhou, Liangcai Leng, Dali Zeng, Guangheng Zhang, Li Zhu, Longbiao Guo, Deyong Ren, Qian Qian, Min Hu

**Affiliations:** 1 State Key Laboratory of Rice Biology and Breeding, China National Rice Research Institute, Hangzhou, 311401 China; 2 National Center for Evaluation of Agricultural Wild Plants (Rice), Department of Plant Genetics and Breeding, China Agricultural University, Beijing, China; 3 College of Advanced Agricultural Sciences, Zhejiang A& F University, Hangzhou, China; 4 National Nanfan Research Institute, Chinese Academy of Agricultural Sciences, Sanya, China

**Keywords:** Rice, Grain size, Salt tolerance

## Abstract

Grain size and stress resistance are key determinants of high and stable yield in rice. However, the genetic and regulatory mechanisms that coordinate these traits remain elusive. Here, we report that *OsHOX24* encoding a HD-ZIP I transcription factor plays a dual role in regulating grain size and salt tolerance. *OsHOX24* promotes grain length and weight by enhancing cell division and expansion in spikelet hulls, while it compromises salt tolerance. Additionally, we demonstrated that OsHOX24 participates in ABA signaling and directly activates *OsPP2C09* transcription by binding to its promoter, thereby jointly regulate grain size and salt tolerance. Finally, haplotype analysis revealed differentiation of *OsHOX24* between *indica* and *japonica*, with the prevalent indica haplotype (Hap1) associated with superior grain size and salt tolerance. Taken together, our study not only provides novel insights into the molecular mechanisms that regulate grain size and salt tolerance, but also offers a valuable haplotype-specific target for breeding high-yielding and salt-resilient rice.

**Supplementary Information:**

The online version contains supplementary material available at 10.1186/s12284-026-00920-w.

## Introduction

Rice (*Oryza sativa*) serves as a fundamental staple crop, sustaining over half of the global population. To meet the rising food demand caused by rapid population growth, it is imperative to develop elite cultivars that integrate high yield and strong stress resilience. However, achieving this synergy remains a formidable challenge between yield and stress tolerance-a phenomenon often characterized by antagonistic pleiotropy or competing metabolic demands (Qian et al. 2016; Bailey-Serres et al. 2019). Deciphering the genetic and molecular basis governing this relationship is essential for engineering the next generation of climate-resilient, high-yielding rice (Wang et al. 2024). Grain size is a determinative component of rice yield and quality and has been a primary selection target during domestication. As a complex quantitative trait, grain size is orchestrated by an intricate network of signaling pathways. Pivotal regulators identified to date include *GS3*, *OsTCP19*, *COR1*, and *OsPP2C09* (Fan et al. 2006; Miao et al. 2020; Xu et al. 2025). Specifically, *GS3* encodes a G-protein γ subunit, in which natural allelic variations account for the divergent grain length observed between *indica* and *japonica* subspecies (Fan et al. 2006). The regulatory landscape further encompasses OsTCP19, which antagonistically regulates grain length and weight by forming a complex with the F-box protein COR1 (Xu et al. 2025). Additionally, the clade A type‑2C protein phosphatase OsPP2C09 integrates growth regulation with abscisic acid (ABA) signaling, functioning as a positive regulator of grain size (Zhou et al. 2015; Miao et al. 2020). Similarly, salt tolerance in rice is a multifaceted quantitative trait controlled by diverse loci, such as *SKC1*, *DST*, *OsMYB106, OsPRR73* (Ren et al. 2005; Huang et al. 2009; Wang et al. 2020; Wei et al. 2021). Although, grain size and salt tolerance have been studied extensively, the molecular mechanisms that coordinate these two traits remain poorly understood.

Abscisic acid (ABA) is a crucial phytohormone regulating plant growth and abiotic stress adaptation (Fujii et al. 2009; Rowe et al. 2016; Zhang et al. 2020). Under salinity, elevated ABA levels confer tolerance via triggering stomatal closure, transcriptional reprogramming, and osmolyte accumulation (Ryu and Cho 2015; Sah et al. 2016; Chen et al. 2022). In the canonical of ABA signaling pathway, PYR/PYL/RCAR receptors perceive ABA and form a complex with PP2Cs, thereby inactivating PP2C activity and releasing the suppression of SnRK2 kinases. Subsequently, activated SnRK2s phosphorylate downstream transcription factors to induce the expression of ABA-responsive genes (Sah et al. 2016). In rice, most PP2Cs negatively regulate ABA signaling and stress tolerance, although many are ABA-inducible. For example, OsPP2C50 and OsPP2C53, two major negative regulators of ABA signaling regarding stomata closing in rice. Similarly, OsPP2C09 promotes growth but suppresses drought tolerance via the ABA pathway (Miao et al. 2020). However, whether OsPP2C09 also functions in salt stress adaptation remains unclear.

The homeodomain-leucine zipper I (HD-ZIP I) family encodes transcription factors with diverse roles in plant development and stress adaptation. In rice, 14 HD-ZIP I members have been identified, and several have been functionally characterized (Agalou et al. 2008). For instance, *OsHOX4* positively confers salt and drought tolerance while positively regulating tiller number (Zhou et al. 2015, 2020). *OsGT1* encodes OsHOX12, which exhibits pleiotropic effects, orchestrating panicle exsertion, plant height, tiller number alongside cold tolerance, often in coordination with its close homolog *OsHOX14* (Gao et al. 2016; Shao et al. 2018; Guo et al. 2025). In addition, *OsHOX22* suppresses drought and salt tolerance via ABA-mediated signaling pathways (Zhang et al. 2012). Despite these advances, the biological functions of many HD-ZIP I members, particularly their potential roles in coordinating the yield and stress traits remain largely unexplored.

Here, we report that *OsHOX24* as a key HD-ZIP I transcription factor, regulates both grain size and salt tolerance. We demonstrated that OsHOX24 participates in ABA signaling, directly binds to the promoter of *OsPP2C09* and activates its expression, consequently influencing grain size and salt tolerance. Further haplotype analysis revealed allelic divergence of *OsHOX24* between *indica* and *japonica*, and identified an elite *indica* haplotype of *OsHOX24* associated with longer grains and improved salt tolerance. Our study provides molecular insight into the genetic coordination of grain size and salt tolerance and highlights allelic variants with potential for developing high‑yielding, salt‑resilient rice cultivars.

## Materials and Methods

### Plant Materials and Growth Conditions

To investigate *OsHOX24* function, all transgenic lines were generated in the background of the rice cultivar Taichung 65 (T65). Mutants of *OsHOX24* and *OsPP2C09* were constructed using the CRISPR/Cas9 system reported by Ma et al. (Ma et al. 2015). The *oshox24*/*ospp2c09* double mutants (*DM-1* and *DM-2*) were generated by crossing the *hox-1* with the *pp2c09-1* and *pp2c09-2*, respectively. All transgenic lines were verified through PCR genotyping, sanger sequencing, and quantitative real-time PCR (RT-qPCR). Homozygous T_2_ or T_3_ generations were utilized for all experiments. Additionally, near-isogenic lines harboring the *OsHOX24*^*−Hap1*^ locus from the cultivar 93–11 within the Nipponbare (*OsHOX24*^*−hap2*^) background were developed via backcrossing and selection from a chromosomal segment substituted line. Experimental plants were grown under standard field management at the Shangzhuang Experimental Station of China Agricultural University (Beijing) and the Hainan Experimental Base. Planting density was 24 cm between rows and 13.8 cm between individual plants.

### Phenotypic Investigation and Statistical Analysis

Grain length and width were determined using an SC-G Automatic Seed Analyzer (Hangzhou Wanshen Detection Technology Co., Ltd., Hangzhou, China). For salt tolerance evaluation, rice seedlings were grown hydroponically under standard conditions for 14 days. Subsequently, the seedlings were subjected to 150 mM NaCl treatments for 7 days, followed by 7 days recovery. Survival rates were quantified upon completion of the recovery phase. To analyze the transcriptional responses, root and shoot tissues were sampled at 0, 1, 3, 6, and 10 h post-treatment with either ABA or salt. All samples were immediately flash-frozen in liquid nitrogen and stored at -80 °C prior to RNA extraction and RT-qPCR analysis.

Dehulled seeds were surface-sterilized and inoculated onto half-strength Murashige and Skoog (1/2 MS) agar medium supplemented with various concentrations of ABA (0, 3, 6, or 10 μM). The seeds were incubated at 28 °C under a controlled photoperiod (14 h light/10 h dark) for 7 days. Germination progress was monitored and documented photographically at 24, 36, 48, 60, 72, 84, and 96 h post-sowing. Germination rates were subsequently calculated based on these observations. For each treatment, three independent biological replicates were performed, with each replicate consisting of 50 seeds each.

Statistical significance was determined using two-tailed Student’s *t*-test for pairwise comparisons or one-way analysis of variance (ANOVA) followed by post-hoc tests for multiple comparisons. All statistical analyses were performed with GraphPad Prism 9.0 (GraphPad Software, San Diego, CA, USA). Graphical representations were generated and refined using Microsoft Office 2016 (Microsoft Corp., Redmond, WA, USA). Data are presented as means ± standard deviations (SDs). The significance thresholds were defined as follows: **p < 0.01 and *p < 0.05.

### Evolutionary Relationships Analysis

To elucidate the evolutionary relationship of OsHOX24 within the rice genome, protein sequences of HD-ZIP I subfamily members were retrieved from public databases as previously described (Agalou et al. 2008). A phylogenetic tree was constructed using MEGA version 7.0 (Kumar et al. 2016) based on the neighbor-joining (NJ) method with 1,000 bootstrap replicates.

### Subcellular Localization and GUS Staining Analysis

To determine the subcellular localization of OsHOX24, the fusion expression vectors p*35S::PROG7-RFP* (a nuclear marker; Hu et al. 2018) and *p35S::HOX24-GFP* were constructed. These constructs were co-transformed into rice protoplasts using the PEG-mediated method as described (Bart et al. 2006). After incubation at 28 °C in the dark for 12 h, GFP and RFP fluorescence signals were visualized using a Leica TCS SP900 confocal laser-scanning microscope (excitation 488 nm and emission 543 nm). For tissue-specific expression analysis, an approximately ~ 2.5-kb promoter fragment was cloned into pCAMBIA1381 to generate *ProHOX24::GUS* and transformed into T65. Various tissues from T_2_-generation plants, including seedling, node, stem, leaf sheath, and young panicles were sampled for analysis. Histochemical GUS staining was conducted using Gusblue kit^@^ (GT0391, Huayueyang, Beijing, China) in accordance with the manufacturer’s instructions.

### RNA Extraction and RT-qPCR

Total RNA was isolated using TRIzol reagent (Invitrogen, USA) and subsequently treated with DNAse I to eliminate residual genomic DNA. First-strand cDNA was synthesized from 1 μg of total RNA using ReverTra Ace® qPCR RT Master Mix with gDNA Remover (FSQ-301, Toyobo Co., Ltd., Osaka, Japan). RT-qPCR was conducted using TOROGreen® qPCR Master Mix (QST-100P/QST-100, TOROIVD, Guangzhou, China). *Actin* was employed as the internal control and relative expression levels were calculated using 2^−ΔΔCt^ method. Each assay included three technical replicates and the sequences of the primers used for RT-qPCR were listed in Supplementary Table S1.

### Scanning Electron Microscopy Analysis

Mature grains were harvested and air-dried to a constant weight. The lemma surfaces were sputter-coated with gold for scanning electron microscopy (Hitachi S-3000N & Quorum PP3000T) to observe grain surface structures. Quantitative image analysis was performed using ImageJ software (version Zulu 13.0.6).

### Transcriptome Analysis

Young panicles (3–5 cm and 5–7 cm) from WT and *hox-1* plants were collected and pooled for RNA extraction. cDNA libraries were constructed and sequenced on the Illumina HiSeq2500 platform (Biomarker Technologies, Beijing). Raw sequencing reads were processed and filtered on the BMKCloud platform (www.biocloud.net) and subsequently aligned to the rice reference genome IRGSP-1.0 (MSU7). Differentially expressed genes (DEGs) were identified using thresholds of |log_2_ (fold change) |≥ 1 and a false discovery rate (FDR) < 0.01. Functional annotation and enrichment analyses were performed on the selected DEGs. Heatmaps were generated from FPKM values using the pheatmap package in R to visualize expression profiles between genotypes.

### Yeast One-Hybrid Assays

To investigate the potential binding of OsHOX24 within the *OsPP2C09* promoter, putative binding regions analysis were performed using PlantPAN (http://plantpan.itps.ncku.edu.tw/). For the Y1H assay, the promoter of *OsPP2C09* was cloned into pLacZi to drive the expression of the *LacZ* reporter gene. The full-length coding sequence (CDS) of *OsHOX24* was inserted into pb42AD vector to generate the AD-HOX24 fusion plasmid. These plasmids were respectively co-transformed into yeast strain EGY48, plated on SD/-Trp/-Ura plates, and incubated at 30 °C for 3 days. Positive clones were transferred to SD/-Trp/-Ura medium containing X-Gal; blue coloration indicated binding of OsHOX24 to the *OsPP2C09* promoter.

### Electrophoretic Mobility Shift Assays

For heterologous protein expression, the CDS of *OsHOX24* was cloned into pET32a vector and transformed into *Escherichia coli* strain Rosetta (DE3). Recombinant His-tagged OsHOX24 protein was induced and subsequently purified using the M5 Ni-Agarose His-Tagged Protein Purification Kit (Mei5bio, MF207), following the manufacturer’s instructions. The purified recombinant proteins were quantified and normalized to a uniform concentration prior to biochemical assays. EMSA was performed using the LightShift™ Chemiluminescent EMSA Kit (Thermo Scientific, 20148) according to the manufacturer’s protocol. Biotin-labelled DNA probes corresponding to *OsPP2C09* promoter regions and unlabeled competitor fragments were synthesized by Beijing Ruibo Company (China).

### Transient Transcriptional Activity Assays

To evaluate the transcriptional regulatory activity of OsHOX24 on the *OsPP2C09* promoter, a ~ 2.5 kb promoter fragment was cloned into the 0800 LUC reporter vector. The construct 35S:OsHOX24 in the pGreenII 62-SK vector served as the effector, with the empty vector as a negative control. For each assay, 10 μg of the reporter plasmid and 10 μg of the effector plasmid were co-transfected into 200 μL of rice protoplasts from the cultivar ZhongHua17 (ZH17). Following a 16 h incubation at 28 °C in the dark, firefly luciferase (LUC) and *Renilla* luciferase (REN) activities were quantified using the Dual-LUC Reporter Assay System (Promega, USA), and the LUC/REN ratio was calculated to evaluate *OsPP2C09* promoter activity.

### Variation, Origin, and Evolution Analysis

Genetic variation within the 2.5-kb genomic region of *OsHOX24*, comprising a 1.0-kb promoter fragment, the coding sequence (CDS), and a 500-bp 3' UTR, were analyzed using the publicly available 3,000 Rice Genomes Project dataset (https://snp-seek.irri.org; Wang et al. 2018). SNPs from this region were extracted and filtered, retaining only biallelic sites with minor allele frequency (MAF) ≥ 0.05 and the missing rate ≤ 20%. Based on the filtered variants, haplotypes were defined and their distributions were compared among major varietal groups.

## Results

### The Subcellular Localization and Expression Patterns of OsHOX24

**Fig. 1:**
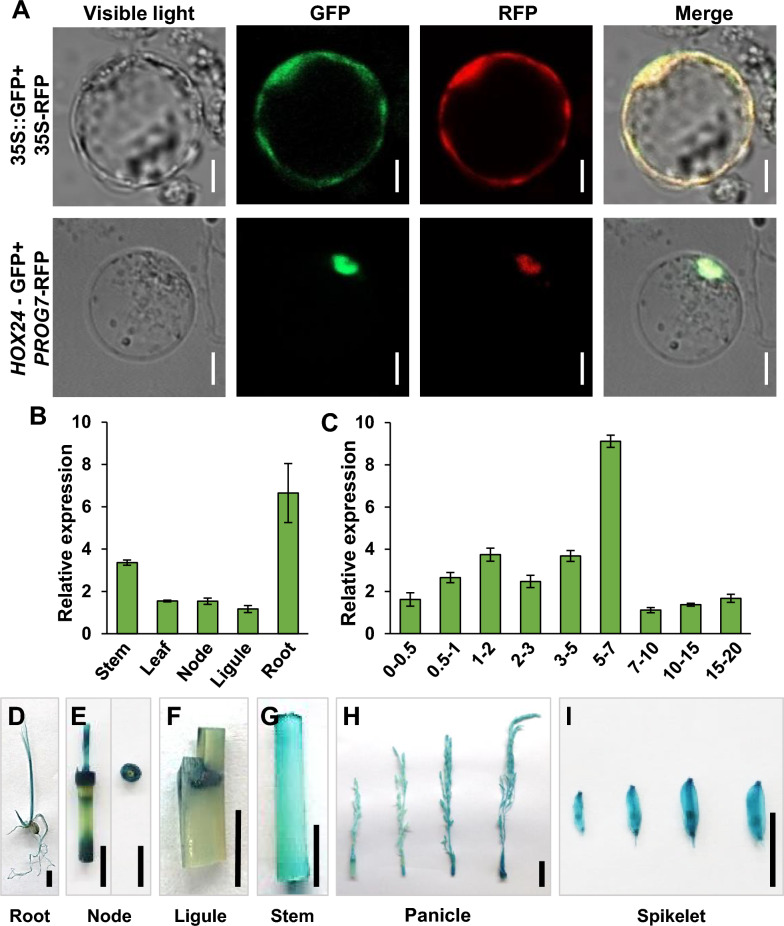
Subcellular localization and expression patterns of *OsHOX24*. **A** Subcellular localization of OsHOX24-GFP and PROG7-RFP fusion proteins in rice protoplasts. The CAMV35Spro::GFP, 35S::RFP, 35S::HOX24-GFP and 35S::PROG7-RFP fusion plasmids were expressed transiently in rice protoplast. Bright, bright-field image; GFP, the GFP channel; RFP, the RFP channel; Merge, all channels merged. Bars = 10 μm. **B**-**C** Expression pattern of *OsHOX24* in T65 is presented as mean SD. (n = 3). **B** Tissue-specific expression patterns of *OsHOX24* in stem, leaf, node, ligule, and root, respectively. **C** Relative expression levels of *OsHOX24* across different developmental stages of panicle. (D-I) Histochemical GUS staining of ProHOX24::GUS in the whole seedling (**D**), node (**E**), ligule (**F**), stem (**G**), developing panicles of 3–7 cm (**H**), spikelet hulls before flowering (**I**), respectively. Bars = 1 cm

*OsHOX24* encodes a transcription factor of the homeodomain-leucine zipper I (HD-ZIP I) family. Transcriptional activation assays in yeast confirmed its transcriptional activity (Fig. S1). To verify its predicted nuclear localization, an OsHOX24-GFP fusion vector and expressed it in rice protoplasts, with the 35S::GFP vector as the control. Confocal microscopy observation revealed that the OsHOX24-GFP fusion protein localized specifically to the nucleus, which was consistent with its role as a transcription factor (Fig. 1A). Spatiotemporal expression analysis by RT-qPCR showed that *OsHOX24* expressed in developing panicles, as well as in stem, leaf, node, ligule**,** and root (Fig. 1B-C). Consistent with the RT-qPCR results, strong GUS staining driven by the *OsHOX24* promoter was observed in multiple tissues, particularly in root and developing panicles. (Fig. 1D-I), thereby suggesting that *OsHOX24* may regulate root and panicle related traits.

### *OsHOX24* Positively Regulates Grain Size Via Cell Proliferation and Expansion

**Fig. 2:**
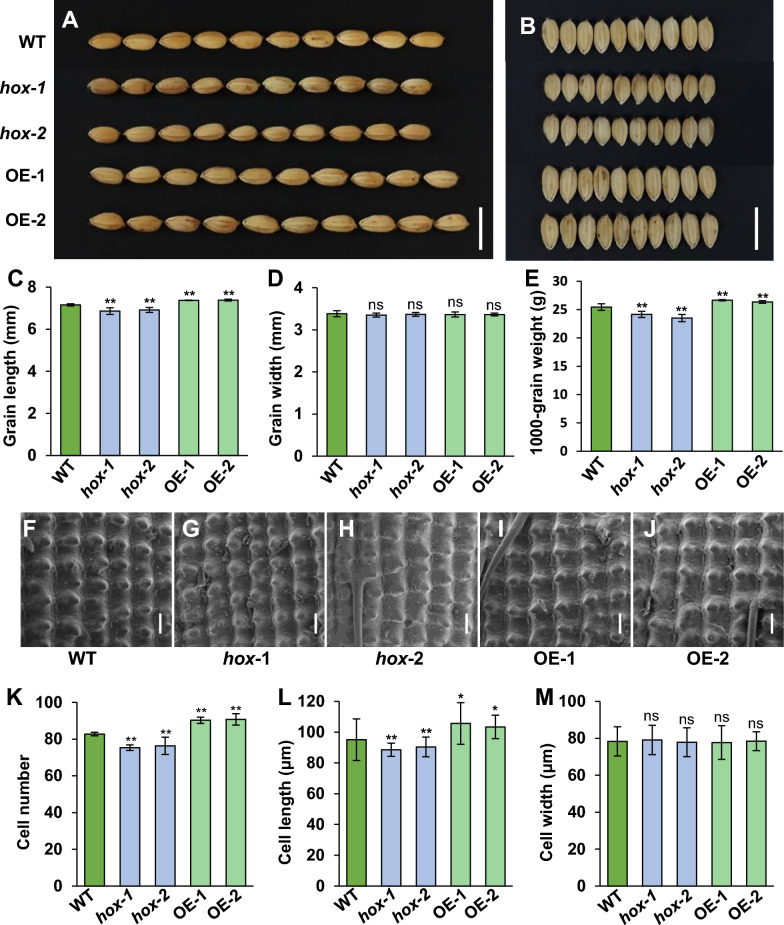
*OsHOX24* positively regulates grain size by modulating cell morphology in rice. **A**, **B** Phenotypic characterization of grains from WT, *hox* mutant and OE lines. Bars = 1 cm. **C**-**E** Statistical analysis of grain length (**C**), grain width (**D**), and 1,000-grain weight (**E**) among WT, hox mutant and OE lines. (n = 30). **F**-**J** Scanning electron microscopy (SEM) of the lemma in mature hulls of WT (F), *hox* mutant (**G**-**H**) and OE lines (**I**-**J**). Bars = 400 μm. (**K**-**M**) Quantification of epidermal cell morphology, including cell length (**K**), cell number (**L**), cell width (**M**) in WT, *hox* mutant lines, and OE lines. (n = 30). Data are presented as mean ± SD, two-tailed Student’s t tests were used (**, *p* < 0.01)

In order to investigate the biological function of *OsHOX24*, we generated knockout mutants in the T65 background using CRISPR/Cas9 technology. Two independent lines (*hox-1* and *hox-2*) were obtained, both of which carry frameshift mutations in the *OsHOX24* gene, thus leading to a disrupted OsHOX24 protein (Fig. S2). Phenotypic analysis revealed that mutant grains were significantly shorter than those of WT, which consequently led to a reduction in thousand-grain weight (TGW) of 5.1% and 7.6%, respectively (Fig. 2A-E). In parallel, we generated *OsHOX24* overexpressing (OE) lines to enable gain-of-function analysis (Fig. S3). By contrast, the overexpressing lines OE-1 and OE-2 produced significantly longer and heavier grains compared with T65, with increases in grain length of 7.6% and 9.6% and in grain weight of 4.74% and 3.53%, respectively (Fig. 2A-E). These results demonstrated that *OsHOX24* functions as a positive regulator of grain size. Further scanning electron microscopy results revealed that mutant lines *hox-1*, *hox-2* had shorter and fewer cells in the spikelet hulls compared with WT, whereas the overexpressing lines OE-1 and OE-2 exhibited longer cells and an increased number of cells (Fig. 2F-M). These results suggest that *OsHOX24* enhances grain length by promoting cell elongation and cell proliferation.

### *OsHOX24* Negatively Regulates Salt Tolerance

**Fig. 3:**
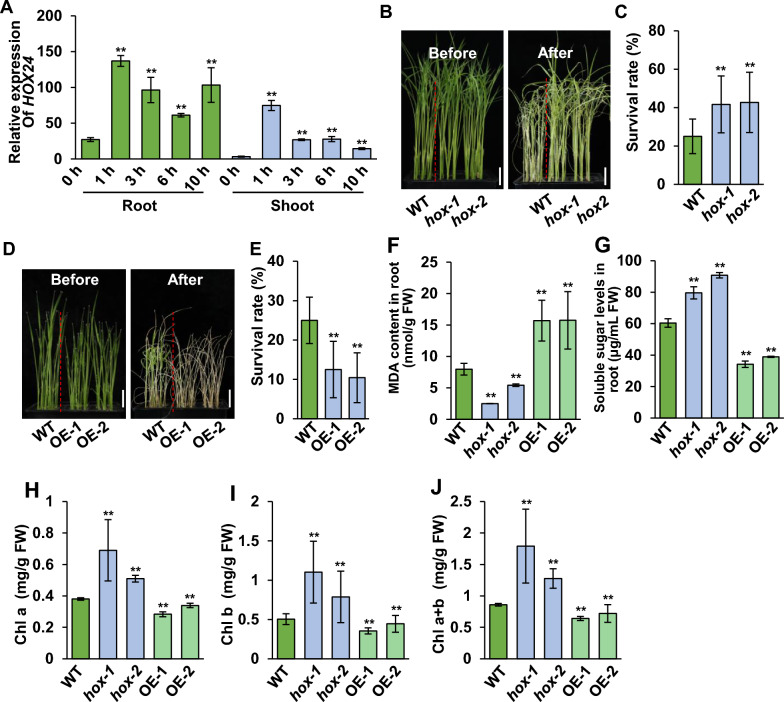
*OsHOX24* negatively regulates salt tolerance in rice. **A** Expression profiling of *OsHOX24* in root and shoot under salt stress. Two-week-old seedlings were treated with 150 mM NaCl and sampled at the indicated time points (0, 1, 3, 6 and 10 h, n = 3). **B**-**E** Salt-stress phenotypes and survival rates of *OsHOX24* transgenic lines. Representative images of WT and *hox* mutants (**B**) and OE lines (**D**) before and after treatment (Bars = 2 cm). Statistical analysis of survival rates for WT, *hox* mutants (**C**) and OE lines (**E**). **F**-**G** Physiological responses in roots under salt stress, including malondialdehyde (MDA) content (**F**), Soluble sugar levels (**G**). **H**-**J** Photosynthetic pigment analysis in shoots, showing contents of chlorophyll a content (**H**), chlorophyll b content (**I**) and Total chlorophyll content (**J**) in shoot. Data represent mean ± SD (n = 3). The significance levels were assessed using Student’s t-test (**, *p* < 0.01)

Phylogenetic analysis identified *OsHOX24* as the closest homolog of *OsHOX22* (Fig. S4), a known mediator of salt stress response (Zhang et al. 2012), prompting us to investigate its functional role in salt response. Consistent with this potential function, RT-qPCR analysis revealed that *OsHOX24* expression was rapidly and significantly induced by salt stress (Fig. 3A). To evaluate its physiological impact, seedlings of WT, *hox* mutant lines, and OE lines were treated with 150 mM NaCl for 7 days, followed by a 7 days recovery period. The survival rates of *hox-1* (41.66%) and *hox-2* (54.76%) were markedly higher than WT (14.85% and 22.22%) (Fig. 3B-C). Conversely, the OE lines were more salt-sensitive with lower survival rates than WT (Fig. 3D-E). Furthermore, physiological parameters, including MDA content, soluble sugar levels, and chlorophyll content (*a*, *b*, and total), were measured after treatment. The *hox* mutants displayed lower MDA and higher soluble sugars/chlorophyll than WT, whereas the OE lines exhibited the opposite pattern (Fig. 3F-J). These physiological data strongly support the contrasting phenotypes observed under salt stress. Collectively, these results demonstrate that *OsHOX24* functions as a negative regulator of salt tolerance in rice.

### OsHOX24 Involves in ABA Signaling

**Fig. 4:**
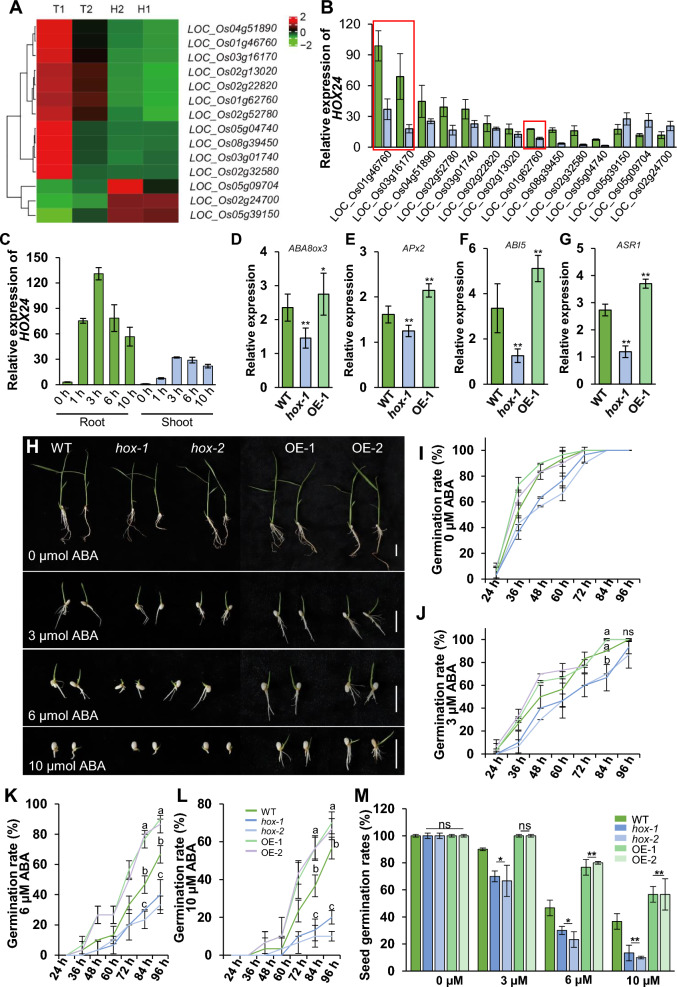
*OsHOX24* modulates ABA signaling and seed germination. **A** Heatmap illustrating relative expression levels (based on FPKM values) of hormone-related differentially expressed genes (DEGs). The color scale represents relative normalized expression levels (green, low; red, high). (B) RT-qPCR validation of representative DEGs shown in A. **C** Expression profiles of *HOX24* in root and shoot of rice seedlings in response to ABA treatment. **D**–**G** Transcript levels of ABA signaling-related genes, including *ABA8ox3* (**D**), *APX2* (**E**), *ABI5* (**F**), and *ASR1* (**G**), in the wild-type (WT), *hox-1* mutant, and OE-1 lines. **H** Germination phenotypes of WT, *hox* mutant, and OE lines following 6 days of exposure to various ABA concentrations. Bars = 1 cm. **I**-**L** Time-course quantitative analysis of seed germination rates under 0 (**I**), 3 (**J**), 6 (**K**) and 10 μΜ (**L**) ABA treatment over a 96-h period. Multiple comparisons were performed among genotypes at 84 h and 96 h. Different letters denote significant differences (*p* < 0.05) followed by Tukey’s multiple comparison test. (**M**) Comparison of seed germination rates at 84 h across different ABA concentrations. (n = 3). Data are presented as mean ± SD. The significance levels were assessed using Student’s t-test (**, *p* < 0.01; *, *p* < 0.05; ns, not significant)

To identify the downstream genes regulated by *OsHOX24*, RNA-sequencing analysis was performed using young panicles from WT and *hox*-*1* plants. A total of 707 differentially expressed genes (DEGs) were detected, comprising 320 upregulated and 387 downregulated genes in *hox-1* compared to WT (Fig. S5A-B). KEGG enrichment analyses revealed that the plant hormone signal transduction pathway was most significantly enriched category (Fig. S5C). Heatmap analysis and RT-qPCR profiling of key genes within this pathway exhibited expression patterns consistent with RNA-seq data (Fig. 4A-B). Notably, the expression of multiple ABA-related genes was significantly altered in *hox-1.* Furthermore, considering that *OsHOX22*, the closest homolog of *OsHOX24*, is strongly induced by salt stress and ABA, these findings suggested that *OsHOX24* play a potential role for in the ABA signaling pathway.

To further investigate the role of *OsHOX24* in ABA signaling, exogenous ABA treatment was performed. RT‑qPCR analysis confirmed that *OsHOX24* expression was strongly induced by ABA (Fig. 4C). We subsequently profiled the expression of core components within the ABA signaling components. All examined genes were downregulated in mutant *hox-1* but upregulated in overexpressed line OE-1 (Fig. 4D-G). Exogenous ABA treatments further validate its role in ABA responses. Compared with the WT, *hox24* mutant lines displayed ABA hypersensitivity, with reduced seed germination rates and shorter primary roots. Conversely, overexpressed lines exhibited accelerated seed germination both with and without exogenous ABA (Fig. 4H-L). Additionally, ABA-mediated inhibition of seed germination and growth occurred in a dosage-dependent manner (Fig. 4M). Collectively, these results demonstrate that *OsHOX24* acts as a key modulator of ABA signaling.

### OsHOX24 Targets *OsPP2C09* to Regulate Grain Size and Salt Tolerance

**Fig. 5:**
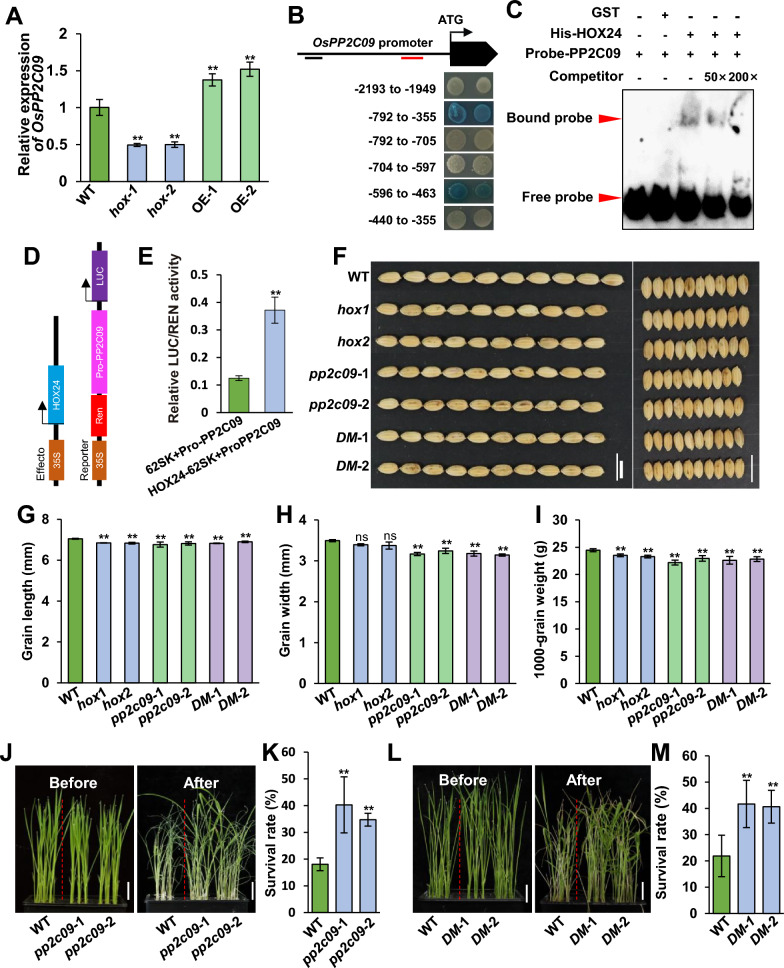
OsHOX24 directly binds to the *OsPP2C09* promoter to regulate grain size and salt tolerance. **A** RT-qPCR analysis of *OsPP2C09* expression levels in WT, *hox* mutant and OE lines. (n = 3). **B** Yeast one-hybrid (Y1H) assay showing the binding of OsHOX24 to the *OsPP2C09* promoter. The promoter sequences were fused with lacZ reporter, black and red line segment indicates the PBS1 and PBS2 motif, respectively. The blue colonies indicated interaction between OsHOX24 and *OsPP2C09* promoter. **C** Electrophoretic mobility shift assay (EMSA) demonstrating that His-OsHOX24 protein directly binds to the *OsPP2C09* promoter. **D**, **E** Dual-luciferase assay in rice protoplasts. Schematic of the effector and reporter constructs (**D**) and the relative LUC/REN activity (**E**) showing OsHOX24-mediated activation of *OsPP2C09* promoter (n = 3). (F-I) Phenotypic characterization (**F**) and statistical analysis of grain length (**G**), width (H), and 1,000-grain weight (**I**) in WT, *hox*, *pp2c09*, and *shox24*/*pp2c09* double mutant lines (*DM*). Bars = 1 cm. **J**-**M** Salt tolerance evaluation of *OsPP2C09* mutant and DM lines. Phenotypes of seedlings before and after 150 mM NaCl treatment (**J**, **L**) and survival rates after recovery (K, M). Bars = 2 cm. Data are presented as mean ± SD, two-tailed Student’s t tests were used (**, *p* < 0.01; ns = not significant)

PP2C family members serve as key ABA-responsive factors involved in plant development and stress response. Among the DEGs, the known ABA-responsive gene OsPP2C09, which encodes a type 2C protein phosphatase and exhibits similar function to OsHOX24 in regulating seed size (Miao et al. 2020), was significantly downregulated in the *hox-1* mutant. This suggested that OsPP2C09 is a putative downstream target of OsHOX24, and we therefore selected it for further analysis. RT-qPCR confirmed that *OsPP2C09* was downregulated in mutant but upregulated in overexpressed lines (Fig. 5A). To examine whether *OsHOX24* directly regulates *OsPP2C09*, we analyzed the ~ 2 kb promoter and identified two putative binding sites (PBS1 and PBS2) recognizing by HD-ZIP I transcription factors (Fig. 5B). Yeast one-hybrid (Y1H) demonstrated that OsHOX24 bound directly to PBS2 (Fig. 5B-C). The electrophoretic mobility shift assays (EMSA) further demonstrated OsHOX24 binds to the PBS2 motif. Dual-luciferase assay result proved that OsHOX24 activated *OsPP2C09* (Fig. 5D-E). Together, these results establish that OsHOX24 directly binds to the promoter of *OsPP2C09* and acts as a positive regulator of its expression.

To assess the biological role of *OsPP2C09*, two independent mutants (*pp2c09-1* and *pp2c09-2*) were generated (Fig. S6). Compared with WT, both *pp2c09* mutants produced smaller seeds with reduced length and width, leading to lower TGW (Fig. 5F-I). To elucidate the genetic relationship between *OsHOX24* and *OsPP2C09*, double mutants (*DM-1*, *DM-2*) were generated. Phenotypic analysis showed that that the double mutants *DM-1* and *DM-2* produced shorter and narrower seeds, resembling the phenotypes of the *pp2c09* single mutant (Fig. 5F–I). These results revealed that *OsPP2C09* acts downstream of *OsHOX24* in regulating seed size. Furthermore, we examined the impact of *OsPP2C09* on salinity tolerance. The *pp2c09* mutants (*pp2c09-1*, 40.8%; *pp2c09-2*, 34.7%) exhibited higher survival rates than WT (Fig. 5J-K), suggesting *OsPP2C09* also acted as a negative regulator of salt tolerance. Notably, both the *pp2c09* single mutant and the *DM*s displayed enhanced salt tolerance, with survival rate of double mutant (40.6%—41.7%) being comparable to the *pp2c09* single mutants (Fig. 5L-M). Collectively, these genetic evidences demonstrated that *OsHOX24* modulates grain development and salt tolerance through an *OsPP2C09*-dependent manner.

### Natural Variations in *OsHOX24 *Locus Enhance Seed Length and Salt Tolerance in *Indica*

**Fig. 6:**
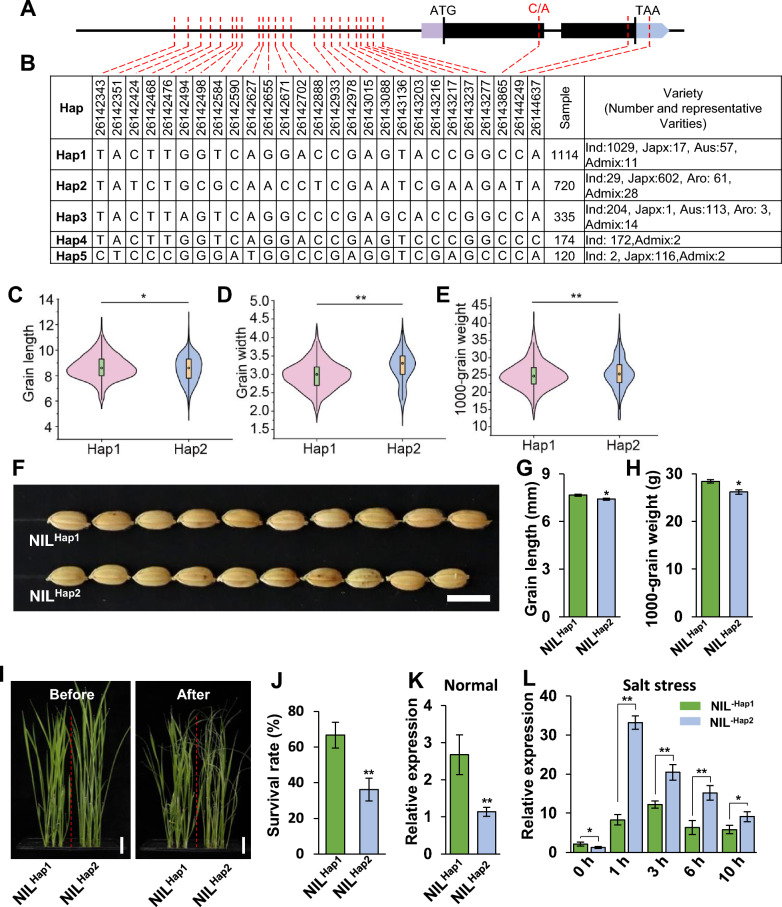
Functional divergence of OsHOX24 haplotypes in regulating grain size and salt tolerance. **A**, **B** Genomic organization and haplotype analysis of the *OsHOX24* across 3,000 rice accessions. Schematic (**A**) shows distribution SNPs (red lines) with a critical non-synonymous variation (C/A) highlighted. **B** Classifies mutation types (pink, synonymous; blue, non-synonymous). **C**-**E** Phenotypic distribution of grain length (**C**), grain width (**D**) and thousand-grain weight (**E**) in varieties carrying Hap1 and Hap2 alleles. (F–H) Phenotypic of Near-Isogenic Lines (NILs) in the Nipponbare (*Hap2*) background. Representative grain images (**F**) and statistical data for grain length (**G**) and 1,000-grain weight (**H**) are shown. Bars = 1 cm. **I**-**J** Salt tolerance assays evaluation of NILs. Phenotypes (**I**) and survival rates (**J**) after 150 mM NaCl treatment. **K**-**L** Expression levels of *OsHOX24-Hap1* and *OsHOX24-Hap2* under control (**K**) and salt-stress (**L**) conditions. Data represent mean ± SD (n = 3). The significance levels were assessed using Student’s t-test (**, *p* < 0.01; *, *p* < 0.05)

To explore the natural allelic variation at the *OsHOX24* locus, we surveyed the Rice SNP-seek Database for polymorphisms across the genome. A total of 27 SNPs were identified within *OsHOX24* genomic region, including 24 in the promoter, 2 within the coding region and 1 within 3' UTR (Fig. 6A-B). Based on these polymorphisms, 2,463 rice accessions, including *indica*, *japonica*, *Aus*, *Bas* and *Admix* were classified into 5 major haplotypes. Among these, Hap1, Hap2, and Hap3 collectively accounted for 88.1% of all accessions (Fig. S7A). In *indica*, 71.7% belonged to Hap1 and 14.2% to Hap3; whereas 81.8% of *japonica* were Hap2 (Fig. S7B-C). These results demonstrate significant haplotype differentiation between two major subspecies, with Hap1 and Hap2 serving as the predominant alleles in *indica* and *japonica*, respectively.

To evaluate the association between the two major *OsHOX24* haplotypes (Hap1 and Hap2) and grain-related traits, we analyzed phenotypic data in the 3 K Rice Genome population. A significant association was observed, accessions harboring Hap1 produced significantly longer and heavier grains compared to those carrying Hap2 (Fig. 6C-E). To establish a causal link and assess the impact on salt tolerance, a pair of near-isogenic lines (NILs), NIL-*HOX24*^*Hap1*^ and NIL-*HOX24*^*Hap2*^, were developed from 93–11(Hap1) and Nipponbare (Hap2) as parents (Fig. S8). Phenotypic characterization confirmed that NIL-*HOX24*^*Hap1*^ produced longer and heavier grains than NIL-*HOX24*^*Hap2*^ (Fig. 6F-H). Furthermore, salt tolerance assays revealed that NIL-*HOX24*^*Hap1*^ (66.7%) had a significantly higher survival rate than NIL-*HOX24*^*Hap2*^ (36.1%) (Fig. 6I-J). Expression analysis revealed that NIL-*HOX24*^*Hap1*^ maintained a higher basal expression level than NIL-*HOX24*^*Hap2*^ under normal growth condition (Fig. 6K). However, under salt stress, although both haplotypes were induced, NIL-*HOX24*^*Hap2*^ exhibited a significantly stronger induction, ultimately reaching higher transcript levels than *HOX24*^*Hap1*^ (Fig. 6L). This divergent expression pattern demonstrates that promoter polymorphisms at the *OsHOX24* locus confer haplotype-specific and environment-dependent transcriptional regulation, which may underlie the observed variations in salt resilience and grain development.

## Discussion

**Fig. 7:**
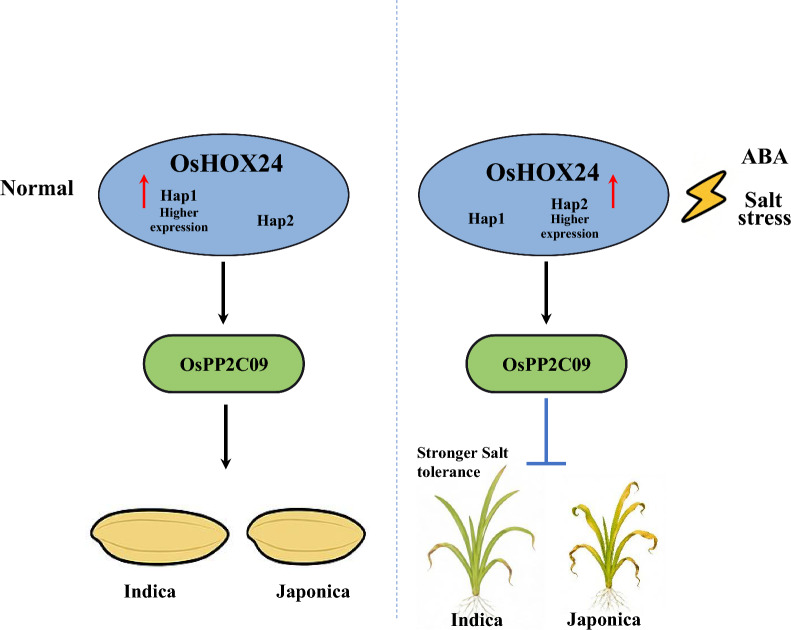
The model of OsHOX24-OsPP2C09 module regulating grain size and salt tolerance. The model illustrates how in *OsHOX24* balances development and stress in rice. Left panel: Under normal growth conditions, the Indica-enriched haplotype (Hap1) of *OsHOX24* exhibits higher basal expression than the *Japonica*-enriched haplotype (Hap2). This leads to increased transactivation of the downstream target *OsPP2C09*, which positively regulates grain size. Right panel: Under salt stress or ABA treatment, *OsHOX24* expression (indicated by the red dashed arrow) is induced. The significantly higher stress-induced expression of the Hap2 allele (predominant in *Japonica*) leads to elevated *OsPP2C09* levels, which subsequently negatively regulates salt tolerance. This regulatory module contributes to the divergent salt-stress sensitivities observed between *Japonica* and *Indica* rice subspecies. Arrows and T-bars indicate positive (activation) and negative (repression) regulation, respectively. The lightning bolt represents salt stress stimuli

Simultaneously improving crop yield and environmental stress tolerance is paramount for sustainable agriculture. Nevertheless, this endeavor is frequently constrained by the inherent negative correlation between these traits. In this study, we systematically characterized the HD-ZIP I transcription factor *OsHOX24* and identified it a pivotal molecular switch governing this balance in rice. Through integrated genetic, molecular, and phenotypic analyses, we demonstrated that *OsHOX24* functions as a pleiotropic regulator that promotes promoting grain size at the expense of salt tolerance. Mechanistically, *OsHOX24* participated in ABA signaling by directly binding to and activating the promoter of *OsPP2C09* to regulate grain size and salt tolerance (Fig. 7). Crucially, we discovered that natural variations at the *OsHOX24* locus have undergone divergent selection, leading to the identification of an elite *indica* allele Hap1. This allele effectively uncouples the yield–stress relationship, conferring both enhanced grain size and robust salt tolerance. These findings not only elucidate the genetic environmental plasticity in rice but also provide a strategic target for breeding climate-resilient crops.

HD-ZIP I transcription factors play critical roles in plant development and environmental plasticity (Ariel et al. 2007). In rice, while several members have been characterized for their function in modulating drought and salt tolerance (Zhang et al. 2012). However, whether any the molecular of HD-ZIP I factor integrates the regulation between seed size (yield) and stress resilience remains largely elusive. Here, we showed *OsHOX24*, as a pleiotropic regulator that antagonistically governs grain development and salt stress response. Knockout mutants exhibited reduced grain length and thousand-grain weight, whereas OE lines produced significantly larger grains, indicating its positive role in grain development. Conversely, OE lines displayed hypersensitivity to salinity, while knockout mutants exhibited enhanced tolerance. Mechanistically, OsHOX24 directly bound to and activated the promoter the type 2C protein phosphatase *OsPP2C09*, forming a function module that regulates grain size and salt tolerance. These findings establish *OsHOX24* as a key genetic rheostat coordinating grain development and salt-stress-associated responses, thereby expanding the functional repertoire of the HD-ZIP I subfamily in crops.

ABA orchestrates seed germination and stress adaptation via the PYR/PYL–PP2C–SnRK2 signaling cascade (Fujii et al., 2007; Cutler et al. 2010; Sah et al. 2016). Consistent with its function as a canonical negative regulator ABA pathway repressor, *OsPP2C09* promotes growth yet compromising drought tolerance (Miao et al. 2020; Min et al. 2021; Kim et al. 2022). In this study, we observed that *OsHOX24* transcription was rapidly induced by ABA, and the encoded protein directly transactivates *OsPP2C09*. Phenotypically, loss-of-function *oshox24* mutants exhibited ABA hypersensitivity, while OE lines displayed enhanced germination and enhanced root elongation under ABA treatment. Transcriptome analysis further showed that core ABA signaling components were significantly downregulated in *oshox24* mutant, but upregulated in overexpression lines. Taken together, these findings suggest that the OsHOX24-OsPP2C09 module acts as a regulatory node that fine-tunes ABA signaling to balance plant growth and stress adaptation.

Salt tolerance in rice involves a range of physiological adjustments, and the control of ionic balance is one of its key components. *SKC1*/*OsHKT1;5*, which encodes an HKT-family Na⁺ transporter, is a well-characterized example that restricts Na⁺ accumulation in shoots and protects rice plants from salt injury (Ren et al. 2005; Kobayashi et al. 2017). The maintenance of K⁺ uptake and distribution are also important under saline conditions, as shown by studies on the K⁺ transporter OsHAK5 (Yang et al. 2014). More recently, transcriptional regulators have also been linked to ion-mediated salt responses; for instance, *OsMYB106* and *OsWRKY72* enhances salt tolerance through SKC1-dependent Na⁺ regulation (Wang et al. 2020; Li et al. 2025). In this study, survival rate, MDA content, soluble sugar content, and chlorophyll content collectively suggest that *OsHOX24* was involved in physiological responses to salt stress. Nevertheless, these measurements are indirect indicators of salt tolerance. However, Na⁺ and K⁺ accumulation and Na⁺/K⁺ ratios were not examined here, we cannot determine whether *OsHOX24* affects ionic balance under salt stress. Future work measuring ion accumulation and ion transporter expression in roots and shoots will provide direct indicators to determine how the OsHOX24–OsPP2C09 module functions in response to salt stress.

Asian cultivated rice (*Oryza sativa*) comprises two major subspecies, *indica* and *japonica*, which exhibit distinct divergences in grain morphology and abiotic stress tolerance. While functional variation in key regulators, such as *GS3*, *GSE5* and *COLD1*, is known to drive these phenotypic differences between *indica* and *japonica* varieties (Fan et al. 2006; Shomura et al. 2008; Ma et al. 2015), the molecular mechanisms balancing yield and adaptation remain largely elusive. Here, our haplotype analysis revealed clear genetic differentiation at the *OsHOX24* locus, with *Hap1* predominating in *indica* and *Hap2* in *japonica*, echoing the subspecific divergence pattern recently reported for *OsMYB8* (Gou et al. 2024). We identified a conditional haplotype advantage driven by distinct transcriptional dynamics. Under optimal growth conditions, the *indica*-prevalent *OsHOX24*^*Hap1*^ maintained higher basal transcriptional activity, promoting larger grains. Conversely, under salt stress, *OsHOX24*^*Hap1*^ exhibited a dampened induction compared to the hypersensitive surge of *OsHOX24*^*Hap2*^, thereby minimizing the survival penalty associated with this negative regulator, a strategy reminiscent of *qRBG1* (Zhang et al. 2024). Therefore, we propose that cis-regulatory variations act as an expression rheostat, fine-tuning *OsHOX24* transcript levels to generate functional divergence. Notably, two coding SNPs in *OsHOX24* resulted in amino acid substitutions (Ala → Asp and Thr → Ile) that altered the protein structure (Fig. S9), potentially contributing to the observed functional divergence between haplotypes. Future studies dissecting how these specific polymorphisms modulate protein–protein interactions or rewire downstream network-will further elucidate the molecular events driving subspecies differentiation in rice.

## Supplementary Information

Additional file1 (PDF 1029 KB)

Additional file2 (XLSX 12 KB)

## Data Availability

No datasets were generated or analysed during the current study.
