# Supplementary material for: OsHOX24 Modulates Grain Size and Salt Tolerance Via OsPP2C09 in Rice
Source: Rice (N Y). 2026 Jun 11;19:57. doi: 10.1186/s12284-026-00920-w (PMC13486418; doi:10.1186/s12284-026-00920-w)
Supplement: Supplementary file 1 [file 12284_2026_920_MOESM1_ESM.pdf]

OsHOX24 modulates grain size and salt tolerance via OsPP2C09 in rice

Bin Gao[†2], Shibo Li[†2], Zhixue Huo[2], Leqin Chang[2], Jing Ning[2], Wei He[2], Liang Luo[2], Mengna Mo[2], Yuxing Zhou[2], Liangcai Leng[2], Dali Zeng[3], Guangheng Zhang[1], Li Zhu[1], Longbiao Guo[1], Deyong Ren[1], Qian Qian[1,4,5]*, Min Hu[1,2]*

9 Supplementary figures:

Figure S1 Transcription activity assay of full-length or truncated OsHOX24 in yeast.
Figure S2 Molecular characterization of rice lines carrying mutations for *OsHOX24*.
Figure S3 Comparison of plant architecture and *OsHOX24* expression levels in OE transgenic lines.
Figure S4 Phylogenetic analysis of the HD-ZIP I subfamily in *Oryza sativa.*
Figure S5 Transcriptomic analysis and validation.
Figure S6 Phenotypic and molecular characterization of rice lines carrying mutations for *OsPP2C09*.
Figure S7 Haplotype analysis of *OsHOX24* and phenotypic association in 3,000 rice accessions.
Figure S8 Graphical genotypes of the recurrent parent Nipponbare and the *OsHOX24*-NIL.
Figure S9 Sequence divergence and structural comparison of *OsHOX24* haplotypes.

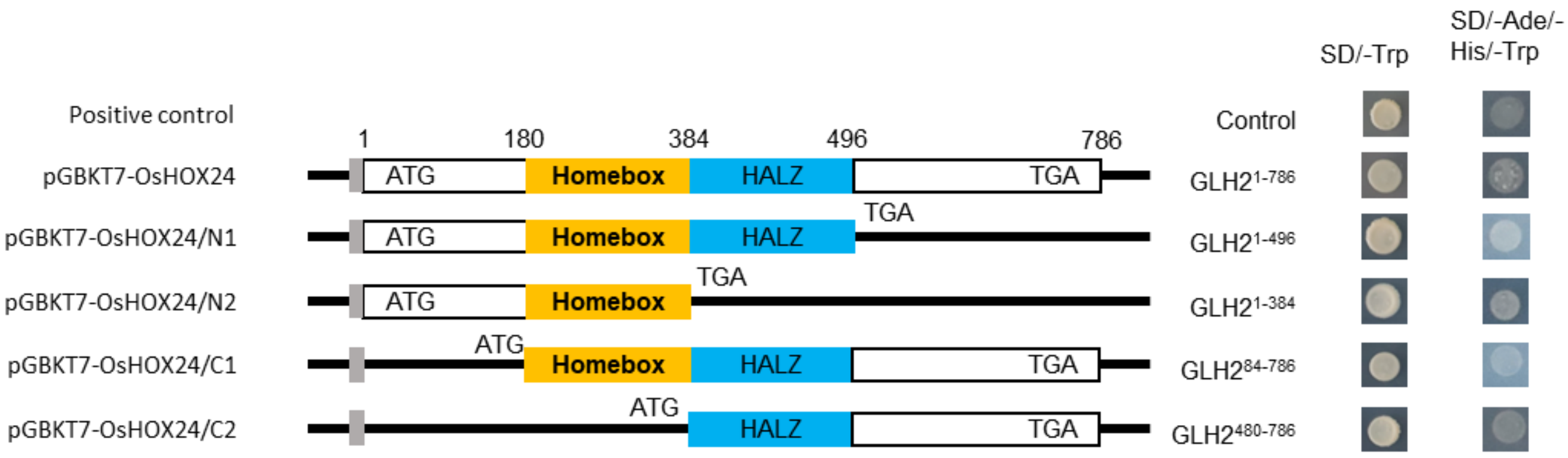

Figure S1 Transcription activity assay of full-length or truncated OsHOX24 in yeast.
pGBKT7-OsHOX24, pGBKT7-OsHOX24/N (1-2), pGBKT7-OsHOX24/C (1-2), had the GAL4 BD (grey) in the pGBKT7 vector fused with sequences encoding full-length, N-terminal or C-terminal, respectively. The transcription factor PROG7 was fused with GAL4 BD as the positive control. The numbers in the boxes indicate base numbering of OsHOX24.

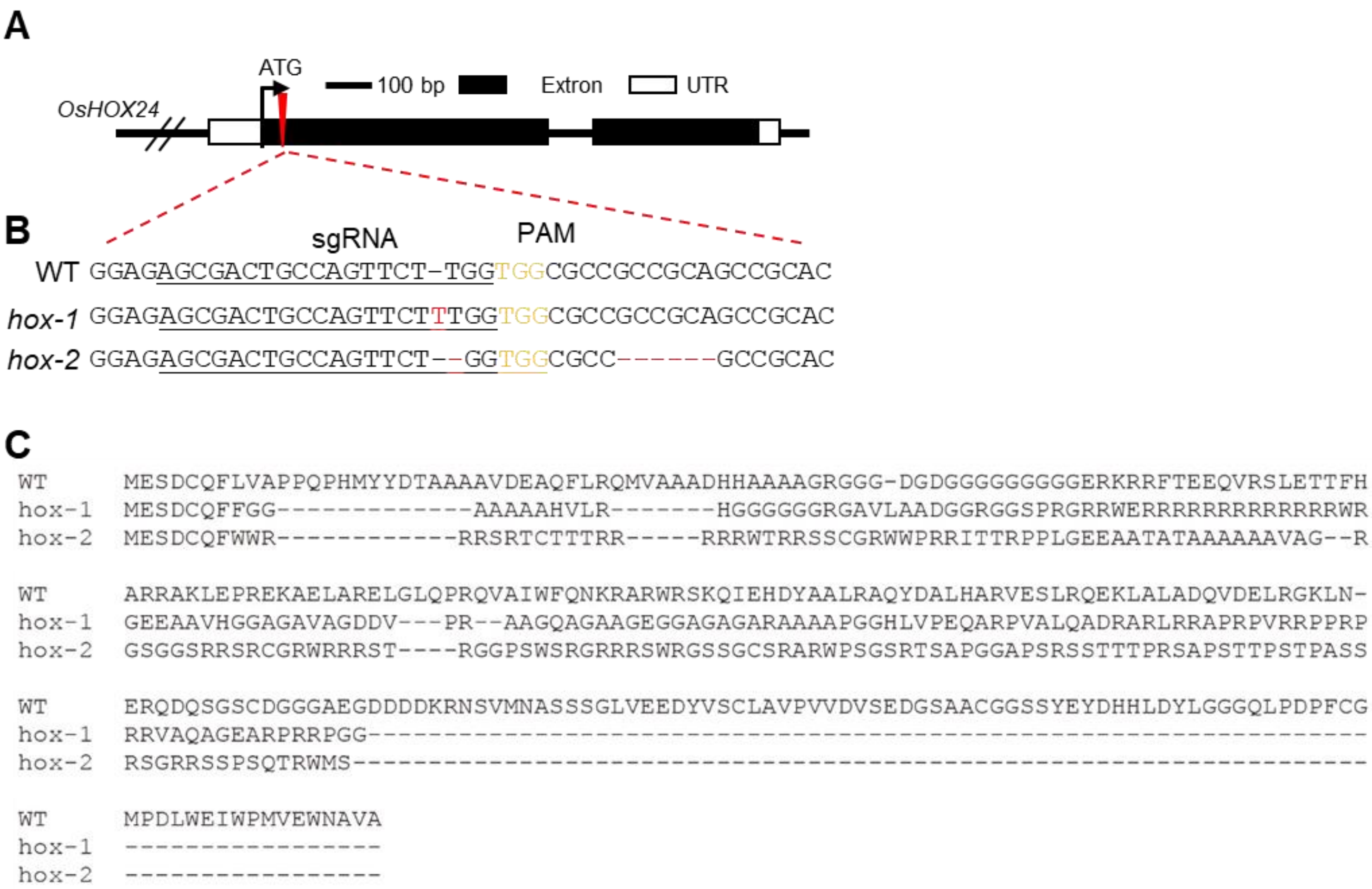

Figure S2 Molecular characterization of rice lines carrying mutations for *OsHOX24*.
(A) The *OsHOX24* exon was edited using CRISPR-Cas9 with T65 as the control. Exon and untranslated regions are represented by black and white boxes, respectively. Bar = 100 bp. (B) Alignment of *OsHOX24* and mutated sequences containing the target sites. Dashed lines indicate deletions. The sgRNA sequence is in underlined and the PAM sequence is marked in red. The NGG site is shown by a red triangle. (C) Amino acid

sequence alignment between OsHOX24 and hox-1 and hox-2 transformants.

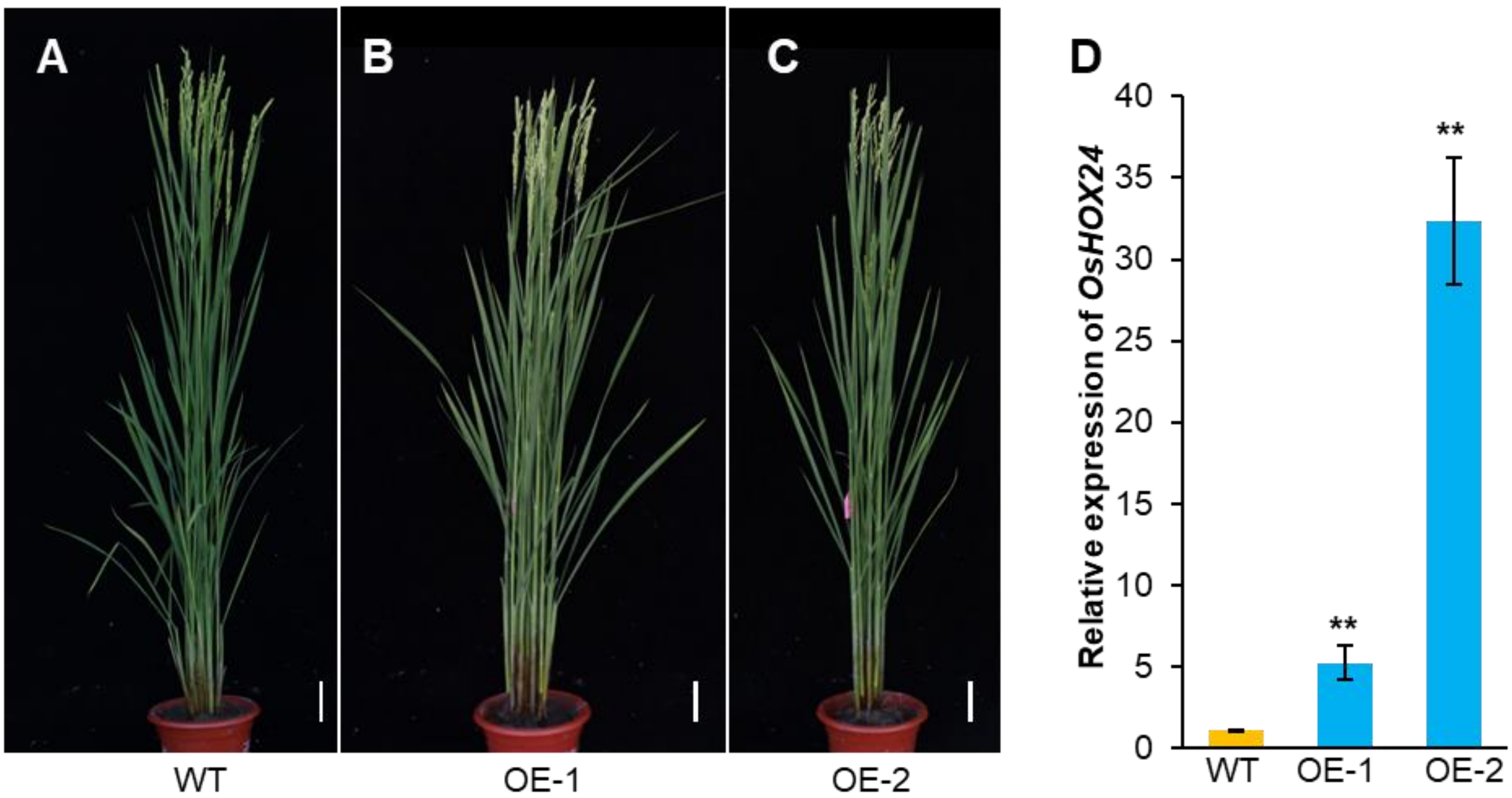

Figure S3 Comparison of plant architecture and *OsHOX24* expression levels in OE transgenic lines. (A–C) Plant architecture of *OsHOX24* OE lines compared with WT. Scale bar = 10 cm. (D) Relative expression levels of *OsHOX24* in OE lines. Data represent means ± SD (n = 3). Statistical significance was assessed using Student's *t*-test (**, $p < 0.01$).

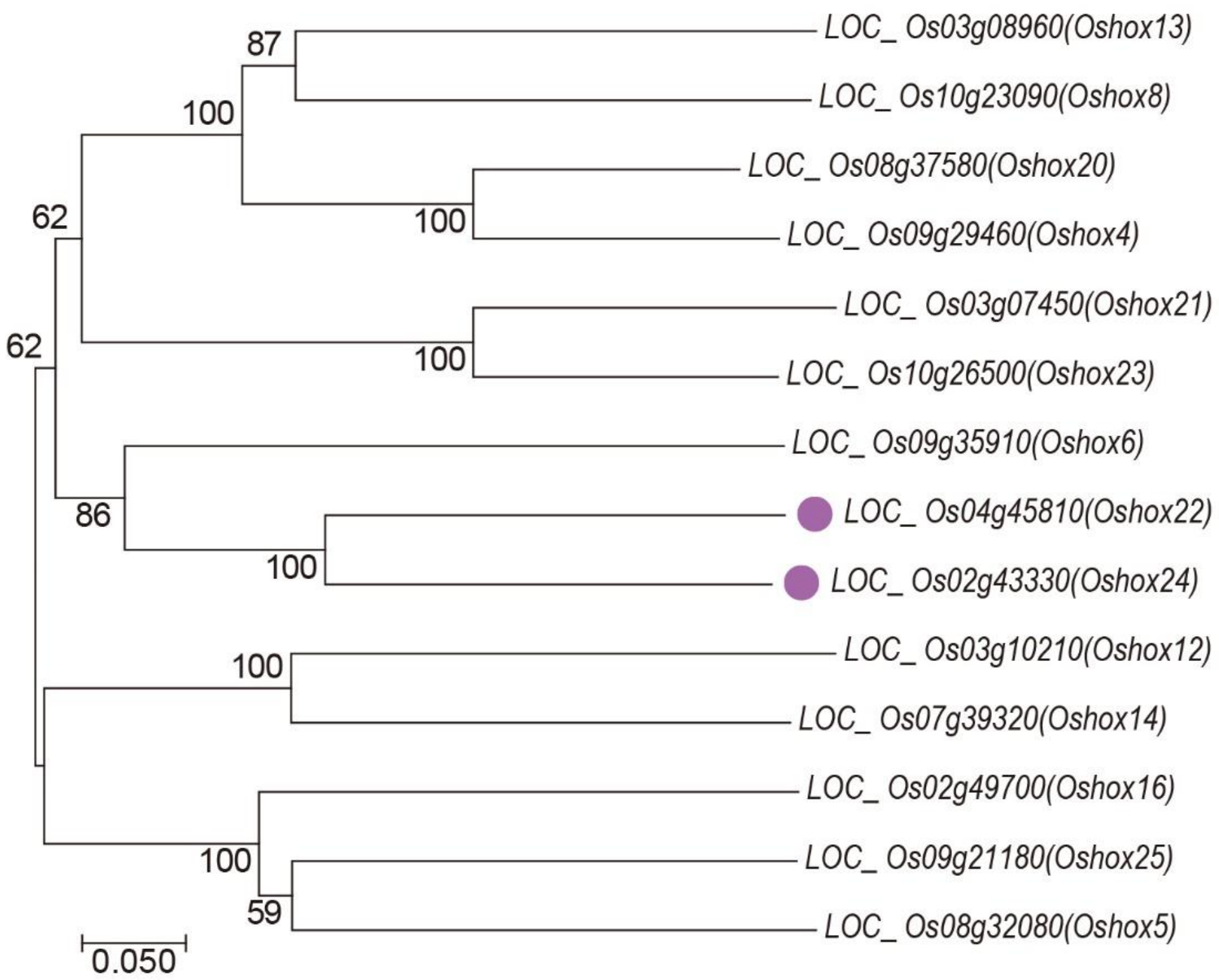

Figure S4 Phylogenetic analysis of the HD-ZIP I subfamily in *Oryza sativa.*

A neighbor-joining phylogenetic tree was constructed based on protein sequences of rice HD-ZIP I genes. OsHOX22 and OsHOX24 are highlighted with purple dots. The scale bar represents an evolutionary distance of 0.050 substitutions per site.

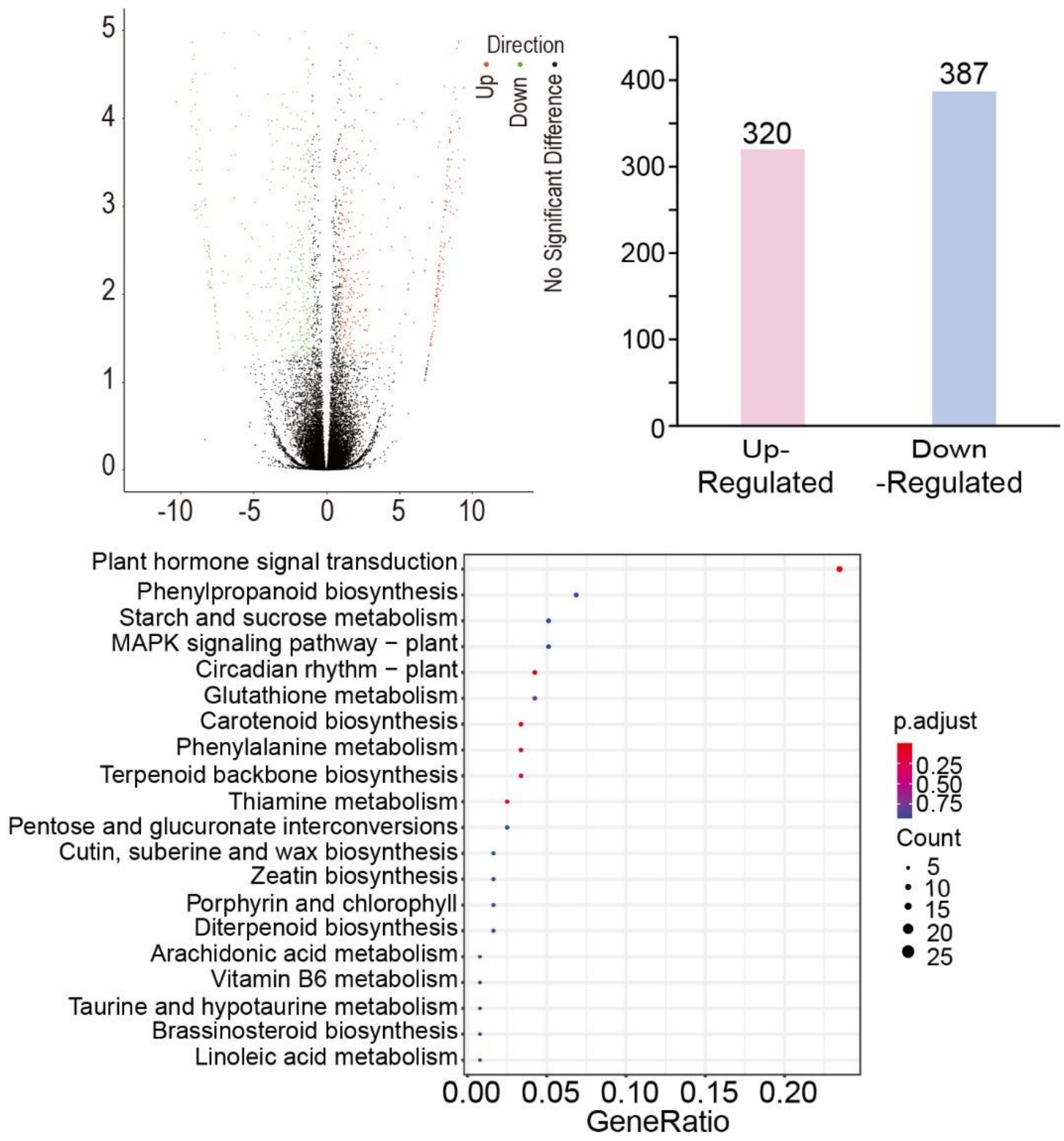

Figure S5 Transcriptomic analysis and validation.

(A) Volcano plot of DEGs. Red and green dots represent significantly up- and down-regulated genes, and black dots indicate no significant difference. (B) Number of DEGs, including 320 up-regulated and 387 down-regulated genes. (C) KEGG enrichment analysis of DEGs. Dot size reflects gene count, and color indicates adjusted *p*-value. Pathways with higher gene ratios and lower adjusted *p*-values are more significantly enriched. (D) Heatmap showing the FPKM values of hormone-related differentially expressed genes (DEGs). The color scale represents relative expression levels (green, low; red, high). (E) Relative expression levels of the DEGs shown in a determined by RT-qPCR. The red boxes highlight genes related to the abscisic acid (ABA) signaling pathway.

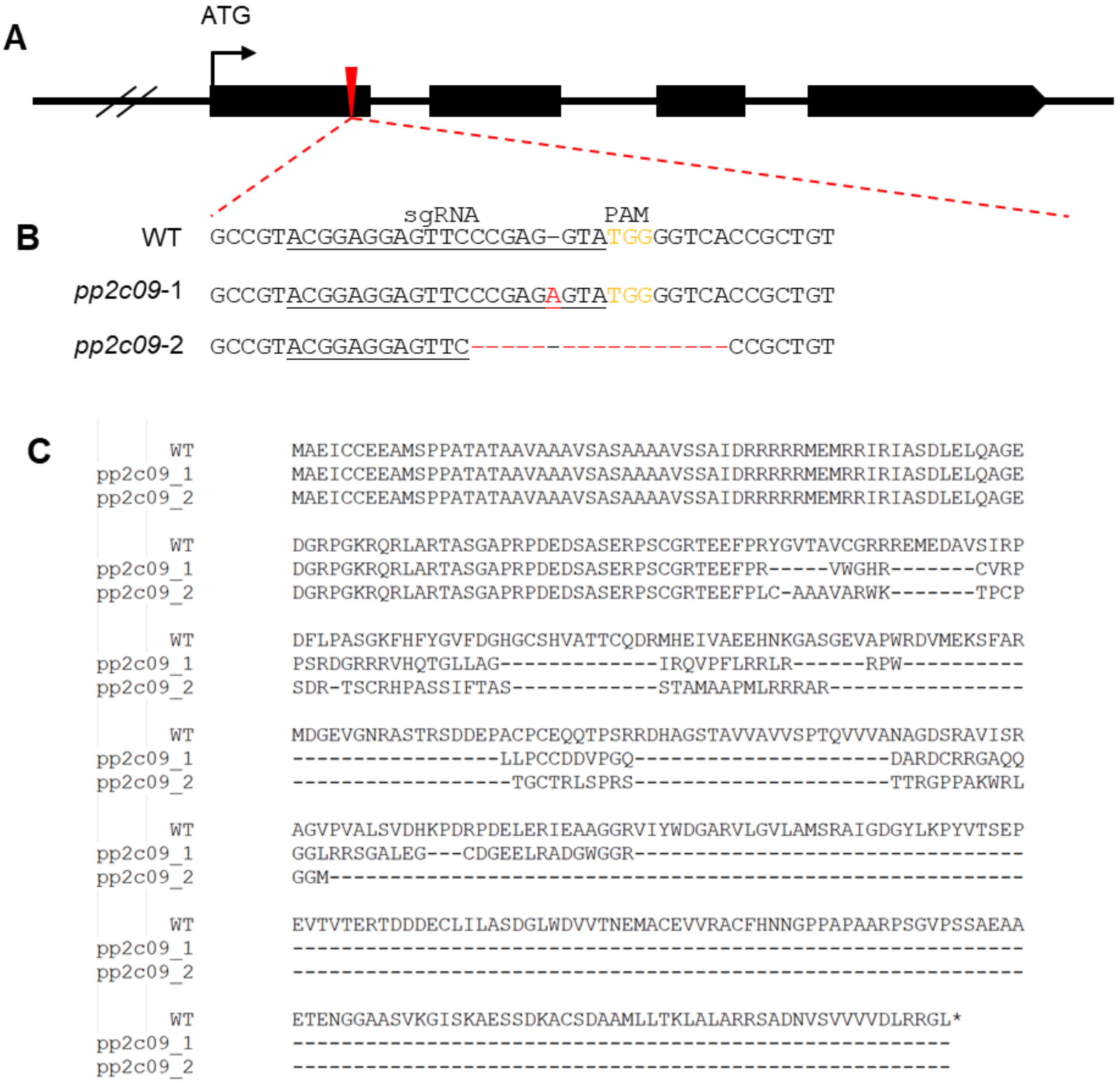

Figure S6 Phenotypic and molecular characterization of rice lines carrying mutations for *OsPP2C09*. (A) The *OsPP2C09* exon was edited using CRISPR-Cas9 with T65 as the control. Exon and untranslated regions are represented by black and white boxes, respectively. Bar = 200 bp. (B) Alignment of *OsPP2C09* and mutated sequences containing the target sites. Dashed lines indicate deletions. The sgRNA sequence is in underlined and the PAM sequence is marked in red. The NGG site is shown by a red triangle. (C) Amino acid sequence alignment between WT, pp2c09-1 and pp2c09-2 transformants.

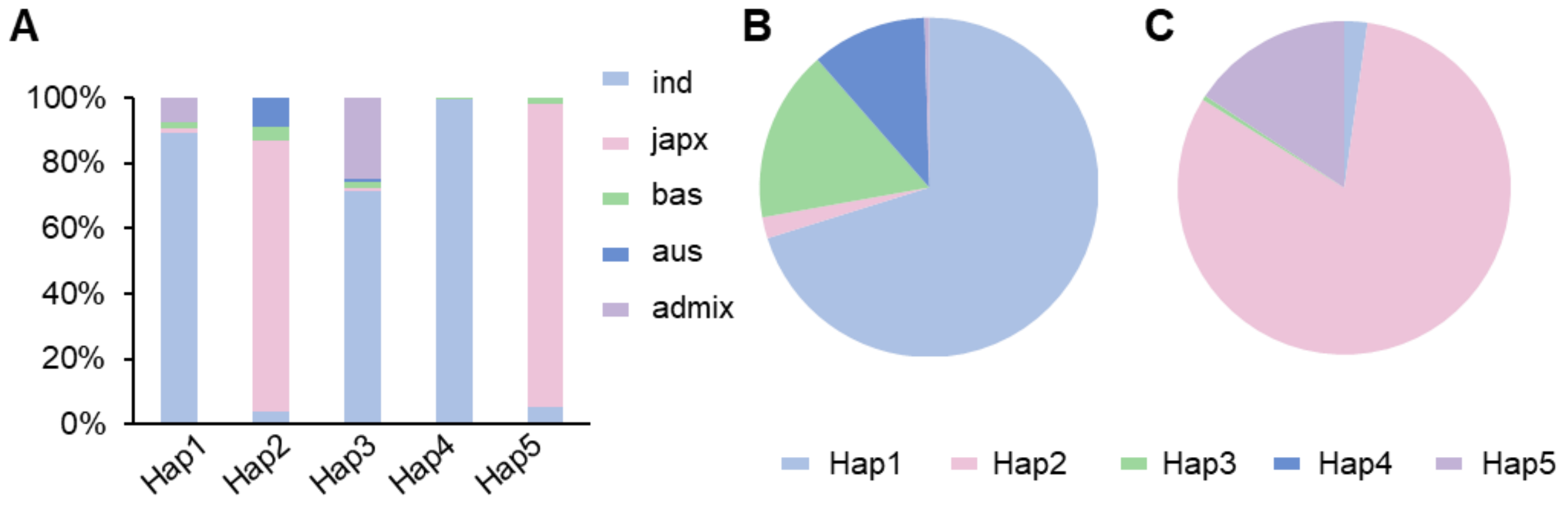

Figure S7 Haplotype analysis of *OsHOX24* and phenotypic association in 3,000 rice accessions.

(A) Stacked bar chart illustrating the composition of rice subpopulations *indica* (ind), *japonica* (japx), *basmati* (bas), *aus*, and *admixed* (admix) across five *OsHOX24* haplotypes (Hap1–Hap5). (B, C) Pie charts showing the proportion of *OsHOX24* Hap1- Hap5 in the *ind* subpopulation (B) and *japonica* subpopulation (C), respectively.

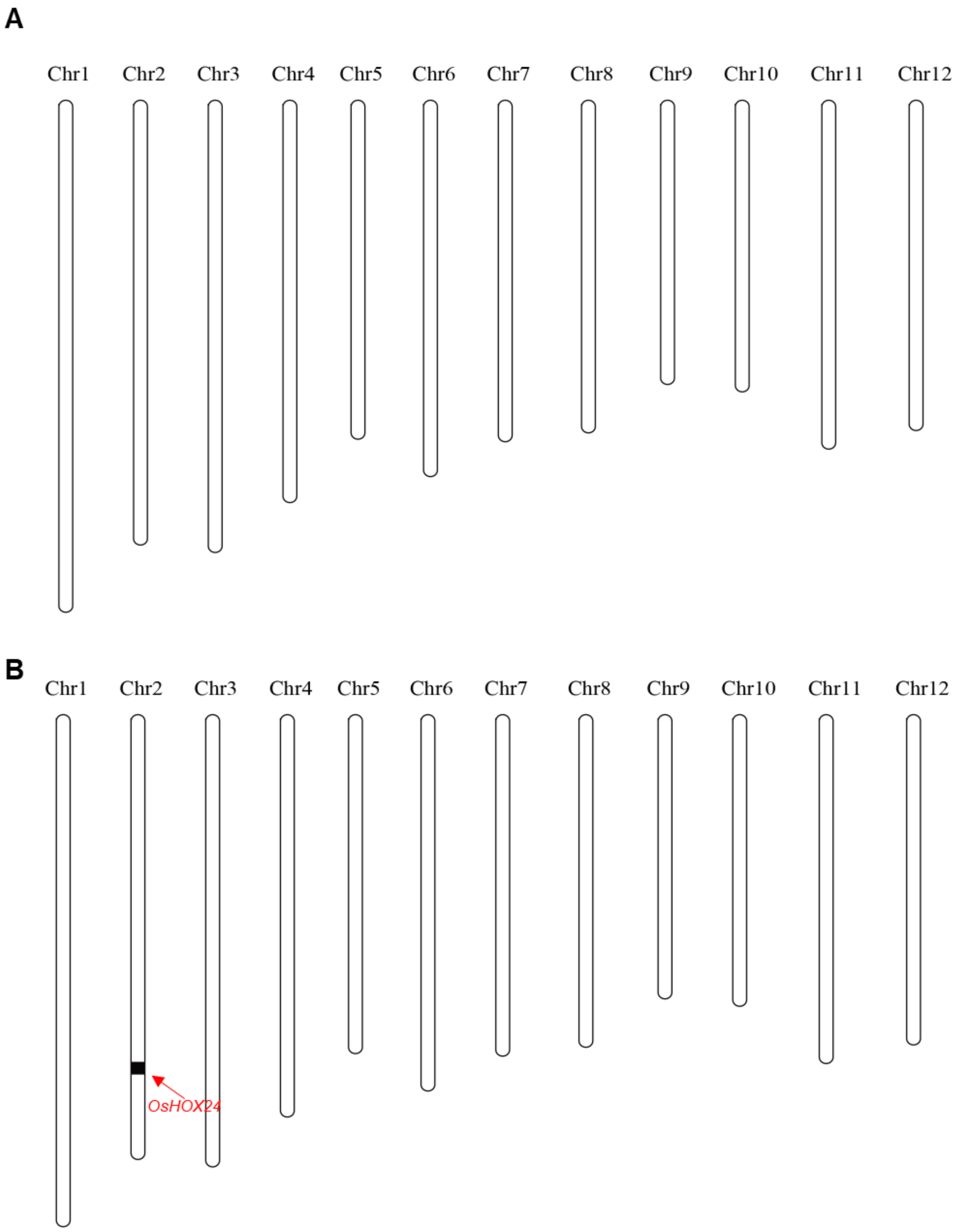

Figure S8 – Graphical genotypes of the recurrent parent Nipponbare and the *OsHOX24*-NIL.
(A) The chromosome complement of the recurrent parent, Nipponbare (NIL-Hap2). (B) The genomic constitution of the *OsHOX24* near-isogenic line (NIL). The black block on chromosome 2 represents the introgressed segment harboring the *OsHOX24* locus, which is indicated by the red arrow.

A

```
HOX-Hap1  MESDCQFLVAPPQPHMYYDTAAAAVDEAQFLRQMVAAADHHAAAAGRGGGDGDGGGGGGG
HOX-Hap2  MESDCQFLVAPPQPHMYYDTAAAAVDEAQFLRQMVAAADHHAAAAGRGGGDGDGGGGGGG
          ************************************************************

HOX-Hap1  GGERKRRFTEEQVRSLETTFHARRAKLEPREKAELARELGLQPRQVAIWFQNKRARWRSK
HOX-Hap2  GGERKRRFTEEQVRSLETTFHARRAKLEPREKAELARELGLQPRQVAIWFQNKRARWRSK
          ************************************************************

HOX-Hap1  QIEHDYAALRAQYDALHARVESLRQEKLALAAQVDELRGKLNERQDQSGSCDGGGAEGDD
HOX-Hap2  QIEHDYAALRAQYDALHARVESLRQEKLALADQVDELRGKLNERQDQSGSCDGGGAEGDD
          ******************************* ****************************

HOX-Hap1  DDKRNSVMNASSSGLVEEDYVSCLAVPVVDVSEDGSAACGGSSYEYDHHLDYLGGGQLPD
HOX-Hap2  DDKRNSVMNASSSGLVEEDYVSCLAVPVVDVSEDGSAACGGSSYEYDHHLDYLGGGQLPD
          ************************************************************

HOX-Hap1  PFCGMPDLWETWPMVEWNAVA
HOX-Hap2  PFCGMPDLWEIWPMVEWNAVA
          ********** **********
```

B

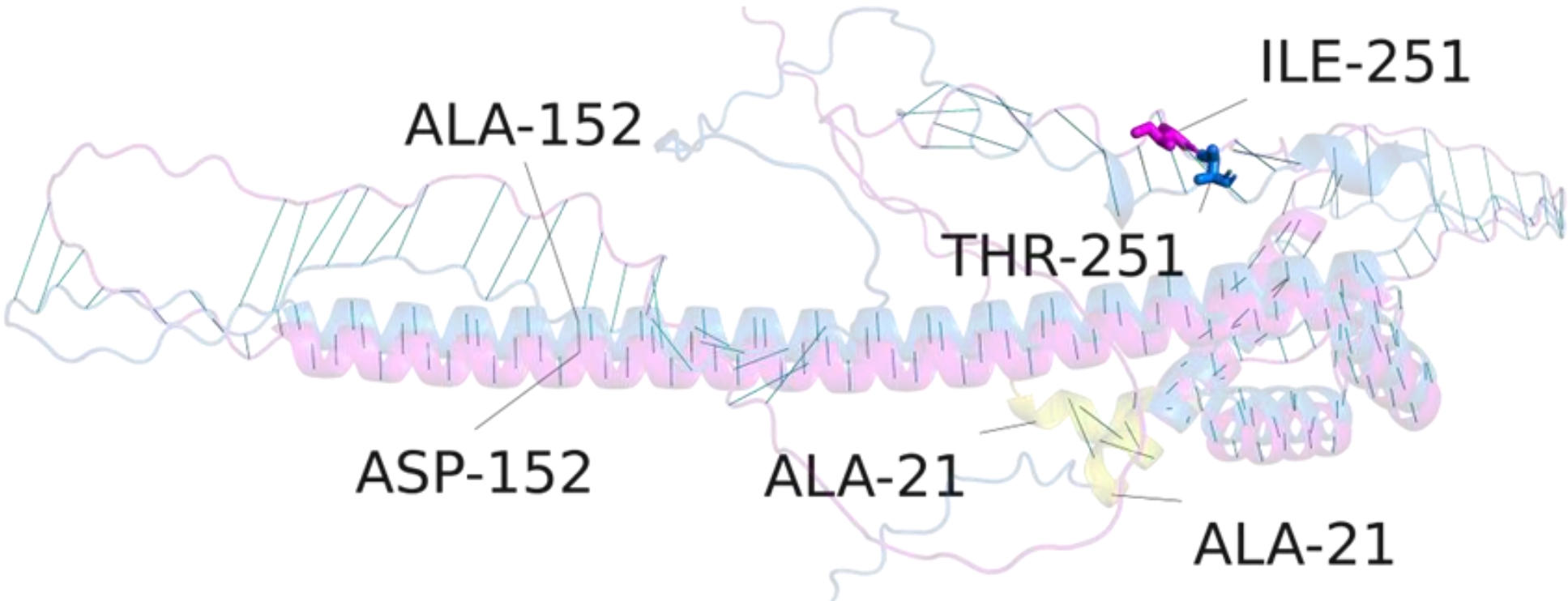

Figure S9 Sequence divergence and structural comparison of OsHOX24 haplotypes.

(A) Pairwise alignment of amino acid sequences. Non-synonymous mutations distinguishing OsHOX24-Hap1 from OsHOX24-Hap2 are framed in red. (B) Superposition of AlphaFold-predicted structures. Key polymorphic residues (Ala152/Asp152 and Thr251/Ile251) are visualized as sticks to demonstrate conformational discrepancies.
